# Koala retrovirus diversity, transmissibility, and disease associations

**DOI:** 10.1186/s12977-020-00541-1

**Published:** 2020-10-02

**Authors:** HaoQiang Zheng, Yi Pan, Shaohua Tang, Geoffrey W. Pye, Cynthia K. Stadler, Larry Vogelnest, Kimberly Vinette Herrin, Bruce A. Rideout, William M. Switzer

**Affiliations:** 1 Laboratory Branch, Division of HIV/AIDS Prevention, Center for Disease Control and Prevention, 1600 Clifton Rd, Atlanta, GA MS G4530329 USA; 2 Quantitative Sciences and Data Management Branch, Division of HIV/AIDS Prevention, Center for Disease Control and Prevention, Atlanta, GA 30329 USA; 3 San Diego Zoo Global, San Diego, CA 92112 USA; 4 Disney’s Animals, Science, and Environment, Bay Lake, FL 32830 USA; 5 Los Angeles Zoo, 5333 Zoo Drive, Los Angeles, CA 90027 USA; 6 Taronga Conservation Society Australia, Taronga Zoo, Mosman, NSW 2088 Australia

**Keywords:** Koala retrovirus, Envelope, Subtypes, Diversity, Pathogenesis, Transmission, Exogenous, Endogenous, Viral load

## Abstract

**Background:**

Koalas are infected with the koala retrovirus (KoRV) that exists as exogenous or endogenous viruses. KoRV is genetically diverse with co-infection with up to ten envelope subtypes (A-J) possible; KoRV-A is the prototype endogenous form. KoRV-B, first found in a small number of koalas with an increased leukemia prevalence at one US zoo, has been associated with other cancers and increased chlamydial disease. To better understand the molecular epidemiology of KoRV variants and the effect of increased viral loads (VLs) on transmissibility and pathogenicity we developed subtype-specific quantitative PCR (qPCR) assays and tested blood and tissue samples from koalas at US zoos (n = 78), two Australian zoos (n = 27) and wild-caught (n = 21) in Australia. We analyzed PCR results with available clinical, demographic, and pedigree data.

**Results:**

All koalas were KoRV-A-infected. A small number of koalas (10.3%) at one US zoo were also infected with non-A subtypes, while a higher non-A subtype prevalence (59.3%) was found in koalas at Australian zoos. Wild koalas from one location were only infected with KoRV-A. We observed a significant association of infection and plasma VLs of non-A subtypes in koalas that died of leukemia/lymphoma and other neoplasias and report cancer diagnoses in KoRV-A-positive animals. Infection and VLs of non-A subtypes was not associated with age or sex. Transmission of non-A subtypes occurred from dam-to-offspring and likely following adult-to-adult contact, but associations with contact type were not evaluated. Brief antiretroviral treatment of one leukemic koala infected with high plasma levels of KoRV-A, -B, and -F did not affect VL or disease progression.

**Conclusions:**

Our results show a significant association of non-A KoRV infection and plasma VLs with leukemia and other cancers. Although we confirm dam-to-offspring transmission of these variants, we also show other routes are possible. Our validated qPCR assays will be useful to further understand KoRV epidemiology and its zoonotic transmission potential for humans exposed to koalas because KoRV can infect human cells.

## Background

The majority of koalas (*Phascolarctos cinereus*) are infected with a gammaretrovirus (koala retrovirus, KoRV) that relatively recently became endogenous and is fixed in the genome of every koala cell, including germ cells, and is transmitted vertically in a Mendelian fashion [1–4]. In contrast, exogenous retroviruses like the human immunodeficiency virus (HIV) are transmitted horizontally and are not in every human cell. The finding of, isolated pockets of KoRV-uninfected koalas however, suggests that the endogenization of KoRV has not yet reached all koala populations [1, 3–6]. Like its closest genetic relative, the gibbon ape leukemia virus (GaLV), KoRV is associated with lymphoid neoplasia [1–3]. KoRV has also been suspected of causing immunosuppressive diseases in koalas resulting in opportunistic infections, especially chlamydiosis [1–3].

In 2015, KoRV genetic diversity was found to be greater than originally believed with heterogeneity in subtypes occurring in the variable region A (VRA, amino acid positions 86–230) of the envelope protein (Env) involved in retrovirus receptor recognition and binding [7, 8]. Since then, studies employing PCR and sequence analysis have identified at least 9 different KoRV variants classified as subtypes A to I, with the first KoRV called subtype A and subtype B was found in a small group of captive koalas with increased neoplasia [7–19]. A subtype J KoRV was reported but phylogenetic analysis with these newer subtypes shows it is clearly a subtype B variant [8, 10, 17]. Not surprisingly for retroviruses, defective genomes have also been reported for KoRV subtypes B, D and E and included variants with *env* deletions [7, 16, 20, 21]. Like KoRV-A, KoRV-B and -E were also shown to infect a broad range of cells, including human cell lines, associated with the ability to utilize different thiamine transport protein 1 (THTR1) or other receptors raising concerns for zoonotic potential to humans [22–25]. One study also demonstrated an upregulation of oncogenes in a human cell line infected with KoRV [25]. Zoonotic transmission of simian retroviruses has been previously documented among persons exposed to nonhuman primates, including HIV that is believed to have originated from a zoonotic transfer from apes and monkeys [26].

Similarly, very little is known about viral loads (VLs) of KoRV variants and how they may affect pathogenicity and transmissibility. Increased retroviral levels, like those reported for HIV and human T-lymphotropic virus type 1 (HTLV-1), are associated with increased pathogenicity and transmissibility [27–29]. Studies to date have reported conflicting results for disease associations with specific KoRV subtypes or had study design limitations to infer disease associations. Early VL studies were done on animals that were either all KoRV-A-positive or of unknown subtype status [18, 30]. Of these nine variants, only KoRV-B has been associated with increased pathogenicity in koalas at the Los Angeles (LA) Zoo with leukemia [22]. KoRV-B infection has also been associated with chlamydial disease in wild koalas in Queensland, Australia [31]. However, these results were based on the presence or absence of KoRV-B in animals with and without chlamydial disease and total VL instead of subtype B-specific VL measurements. A newer study showed a possible association of increased KoRV-A pVLs in female koalas with chlamydial disease but plasma KoRV-A levels were not determined and KoRV-B was not found in this large study population [13]. Quigley et al. have also reported a possible association of neoplasia and chlamydial disease in wild koalas with KoRV-B infection in a small number of koalas but viral loads were not measured [14]. A longitudinal study by this group did not find an association of KoRV subtype with chlamydial disease and found higher expression of subtype D in healthy, wild koalas [15]. Another recent study also failed to find an association of KoRV subtype with neoplasia or chlamydiosis, though only small numbers of koalas with sufficient plasma RNA were examined and standard qPCR methods were not used to evaluate disease associations with subtype VLs [16]. A new report found elevated VLs in wild koalas with neoplasia compared to other diseases but only found a positive association of plasma VLs in koalas from South Australia with chlamydiosis but not in koalas from Queensland [32]. This latter study did not examine disease associations by KoRV subtype. Hence, the clinical significance of non-A, non-B subtypes and correlations with VL is currently limited.

Less is known about the transmissibility of KoRV subtypes among koalas. The original study that identified KoRV-B reported dam-to-joey transmission of KoRV-B but only small numbers of captive koalas were studied at one zoo [22]. While these results were supported by another study with a larger number of koalas [14], two recent studies identified discordant dam-to-joey transmission results [19, 33]. The first study reported transmission in captivity of subtype C but not B to a joey from the KoRV-B and -C-infected parents [19]. The second study found two dam-joey pairs in wild koalas for which the dam was KoRV-A-positive but the joeys were KoRV-negative and three pairs for which the joeys were KoRV-A-positive and the dams were KoRV-negative suggesting KoRV-A may be exogenously transmitted in this population [33].

To better understand the distribution, transmissibility, and pathogenicity of KoRV variants, we developed and validated KoRV generic and subtype-specific qPCR assays to detect and quantify these novel subtypes. We applied these new tools to many zoo and wild koalas to determine their geographic and inter-koala distribution and VLs. We also tested biospecimens from zoo koalas with and without disease to evaluate potential associations of KoRV genotype and titers with disease. By using koala pedigree records we evaluated transmission of KoRV subtypes from infected parents to their offspring. Our molecular epidemiology findings will have importance for koala management and for biosafety practices in persons exposed to koalas to reduce zoonotic risks.

## Materials and methods

### Koala specimen collection and processing

EDTA-treated blood specimens for koalas at U.S zoos were opportunistically collected and processed within 24 h. Tissue specimens from U.S. zoo koalas, including spleen, lymph node, liver, heart, kidney, brain, thymus, intestine, lungs, muscle, skin, nodules, and/or bone marrow were obtained at necropsy following recent death or from specimen biobank archives. Archived plasma, and peripheral blood mononuclear cells (PBMCs) or buffy coats, were also available from three koala populations in Australia: (1) Taronga Zoo in Sydney, New South Wales, (2) a Queensland zoo that wished to remain anonymous, and (3) wild koalas from St. Bees Island in Australia that were available from a San Diego Zoo study in collaboration with the University of Queensland. Animal records indicate all koalas in this study are Queensland koalas (*P. c. adustus*). Koala pedigrees were available from the 2017 North American Regional Studbook prepared by the San Diego Zoo and from breeding records kept at the Taronga Zoo. The pedigrees were used to investigate KoRV transmissibility in parent–offspring groups. Longitudinal blood specimens were available from eight koalas at six zoos to assess changes in VLs and subtypes over time. Our study followed the Policy Guide for Conducting Scientific Studies and was approved by the Institutional Animal Care and Use Committee (IACUC) at each zoo.

Blood specimens were processed for plasma and PBMCs using ficoll-hypaque and centrifugation and then stored at − 80 °C. Tissue specimens were collected at necropsy and frozen at − 80 °C and were thawed on ice and homogenized with a Bullet Blender using 0.5 mm zirconium oxide beads (Next Advance, Averill Park, NY). Genomic DNA (gDNA) from PBMCs or tissue specimens were extracted using a QIAamp DNA Mini kit (QIAGEN, Valencia, CA) following manufacturer’s instructions and stored at − 20 °C until testing. RNA was extracted from plasma and tissues using a QIAamp Viral RNA Mini kit (QIAGEN, Valencia, CA) following the manufacturer’s instructions but modified by replacing tRNA in the kit with 10 ug of Dextran. Dextran as a nucleic acid carrier, in contrast to tRNA, does not affect spectrophotometric measurements of nucleic acids at ODs of 260 and 280 nm. RNA samples were treated with DNase I using a DNA-free kit (Thermo Fisher Scientific, Grand Island, NY) to remove any contaminating DNA and tested immediately or stored at − 80 °C. Nucleic acid quality and concentrations were determined using a NanoDrop One microvolume spectrophotometer (ThermoFisher Scientific) that is calibrated at least annually.

### KoRV generic and subtype-specific and koala β-actin qPCR assay design and validation

Available complete and near complete KoRV *env* nucleotide sequences at the time were aligned using Clustal W in MEGA v7 to identify regions of identity and dissimilarity for design of generic and type-specific primers, respectively. The KoRV type-specific primers and probes are located in variable region A in *env* (pos 258-413 in KoRV-A, GenBank # AF151794), which shares 44–70% intersubtype nucleotide identity (Additional file 1: Table S1). The generic primers are located in the highly conserved transmembrane region of *env*. The DNA plasmids of entire *env* sequences for subtypes A (1980-bp), B (2001-bp), E (2010-bp) and F (1998-bp) were initially used in developing the generic and specific qPCRs. For better quantification and comparison of KoRV copy numbers between assays, and for simplifying the qPCR systems, a mosaic plasmid was synthesized by Integrated DNA Technologies (IDT, Coralville, Iowa) to include tandem koala β-actin (GenBank # DQ058212) and generic and subtype-specific (KoRV-A, -B, -E, -F, and -J) *env* sequences (GenBank numbers AF151794, NC_021704, AB822553) as a unique standard control for all seven qPCR assays. KoRV-E and -F *env* sequences were kindly provided by Xu et al*.* prior to publication [7]. To generically detect KoRV-A-J we designed two reverse primers (5′-TGG GAG GTC CTT GTY CTG CGA GGA-3′ and 5′-CAG GGA GAC CTT GTA CTA CAA GGA C-3′) for use in the generic qPCR assay (Additional file 1: Table S1). Subtype-specific detection of subtypes C, D and G-I were not included in our study since they were discovered after completion of our assay validation [8, 10].

Optimal conditions for the qPCR assays included 95 °C for 9 min, 95 °C for 15 s, 62 °C (koala β-actin, KoRV-A and -B) or 68 °C (KoRV-E, -F and -J) for 30 s for 55 cycles using a Bio-Rad CFX96 instrument (Hercules, CA) and the AmpliTaq Gold polymerase (Life Technologies, Grand Island, NY). Ten microliters of RNA extract equivalent to 50 ul plasma or 200 ng tissue was used for KoRV viral and β-actin RNA testing, and 200 ng gDNA was used for proviral and β-actin DNA testing. Koala β-actin DNA testing was used to confirm the quality of the extracted DNA and was not used to normalize DNA pVLs since it is not a single-copy housekeeping gene and studies have shown β-actin levels can vary widely in animals depending on various factors, including cellular activation [34, 35]. Proviral loads were quantified by the number of KoRV copies per ug of DNA, while plasma VLs were quantified by the number of KoRV copies per mL of plasma. The sensitivity of each qPCR assay was evaluated by spiking of diluted synthetic mosaic plasmid into a background of human nucleic acids. Ten replicates of each dilution containing 10, 5, and 1 copies of the mosaic plasmid were tested to determine the reproducibility of assay sensitivity and the calculated detection limits were applied as cut-offs for specimen testing.

### Statistical analyses

Log10 VLs for KoRV-A, -B, -E, -F and -J in both PBMCs and plasma from whole blood samples, and various tissues were analyzed. Descriptive analysis included the mean, median, standard deviation and range of each KoRV subtype VL. Age at testing, final health status classification for each koala (alive, leukemia-lymphoma, other cancers, and other causes of death), sex (female, male and joey) and location (Australian zoo, US zoo, and wild) were included in our analysis for investigating potential associations between KoRV VLs as a continuous outcome and these koala characteristics. As KoRV VLs are subject to an assay detection limit, the Tobit model with random effects was applied to perform the analysis, adjusting for the repeated VL records from animals with multiple specimens. The Tobit model refers to a class of regression models in which the observed range of the dependent variable is censored in some way [36]. In further analyses, leukemia-lymphoma and other cancers were combined into one category. Statistical analyses were performed with SAS v9.4. Box and whisker plots were prepared with igraph in R (https://igraph.org/r/).

## Results

### Koala study population

In total, 126 koalas were available for our study, of which 27 (21.4%) were from two Australian zoos (24 were from Taronga Zoo and 3 from a Queensland zoo), 78 (61.9%) were from ten US zoos, and 21 (16.7%) were wild-caught at St. Bees Island (Table 1). Age for the wild koalas was estimated from their physical characteristics, including length and comparisons with their physical characteristics at first capture 8–13 years ago. Using these estimates and animal records for the zoo koalas, the mean age was 6.2 years old (yo) with a median of 5 yo and 25th and 75th percentiles of 2 and 9 yo, respectively. There was almost an equal number of females (n = 56, 44.4%) and males (n = 58, 46.0%) and 12 (9.5%) joeys. Koalas were considered joeys if they were younger than or equal to 12 months old, the age when they are considered fully weaned. Among the 126 koalas, 66 (52.4%) were alive at the conclusion of the study and generally healthy, 16 (12.7%) had died from leukemia-lymphoma, 7 (5.6%) had died from other cancers (osteoma, metastatic sarcoma, osteosarcoma, hemangiosarcoma, and mammary carcinoma), and 37 (29.4%) had died from other causes, including anemia, old age, euthanasia, acute respiratory illness, pulmonary interstitial fibrosis, intestinal volvulus, torsion, intussusception, early young pouch death, or the cause was not determined (Table 1). Chlamydiosis was not reported for any koala.

**Table 1:** Descriptive characteristics of 126 koalas in the study population

Category	US zoos (%)	Australian zoos (%)	St. Bees Island (wild) (%)	Total (%)
Sex				
Male	38 (48.7)	12 (44.4)	8 (38.1)	58 (46.0)
Female	29 (37.2)	14 (51.9)	13 (61.9)	56 (44.4)
Joey	11 (14.1)	1 (3.7)	0 (0)	12 (9.5)
Health status classification^a^				
Healthy	29 (37.2)	16 (59.3)	21 (100)	66 (52.4)
Leukemia-lymphoma	14 (17.9)	2 (7.4)	0 (0)	16 (12.7)
Other cancers	5 (6.4)	2 (7.4)	0 (0)	7 (5.6)
Other causes	30 (38.5)	7 (25.9)	0 (0)	37 (29.4)
Total	78 (61.9%)	27 (21.4%)	21 (16.7%)	126

^a^Causes of death at study conclusion are listed for other than alive. Other cancers included osteoma, metastatic sarcoma, osteosarcoma, hemangiosarcoma, and mammary carcinoma. Other causes of death included anemia, old age, euthanasia, acute respiratory illness, intestinal volvulus, torsion, intussusception, pulmonary interstitial fibrosis, unknown, and early young pouch death. The most common lesion in koalas that were classified with death at old age was degenerative joint disease

Blood specimens were available from 92 adult koalas (45 males, 47 females) and from 8 joeys with 118 total blood specimens tested when including multiple specimens from eight koalas collected longitudinally. Only tissues (skin, and/or brain and muscle) were available for two joeys that died while in the pouch and only liver and spleen tissues were available for a joey that died from anemia. Multiple tissues (spleen, lymph node, bone marrow, intestine, liver, lungs, nodules, thymus, heart, kidney, and/or brain) were also available from 28 adults at two US zoos for a total of 77 tissue specimens tested. Of these 28 adult koalas, five also had blood specimens available.

### qPCR assay validation

The KoRV generic and type-specific and the koala β–actin assay could each reliably detect 10 copies of DNA or RNA targets/reaction in each replicate. Each assay could detect five copies of DNA or RNA target/reaction except the KoRV-B and KoRV-E RNA specific tests that only detected 90% and 70%, respectively, of the replicates containing five DNA or RNA copies per/reaction. Hence, the assay cutoff for generic and KoRV subtype-specific detection was between 5–10 DNA and RNA copies/reaction. The koala β–actin assay could detect 90% of the DNA and RNA replicates with one copy/reaction and 100% of the DNA and RNA replicates at five copies/reaction, and we thus set the cutoff for that assay at 1–5 copies/reaction.

The linear dynamic range and specificity of each qPCR assay was measured by testing ten replicates of ten-fold dilutions (10^0^ to 10^7^) of the KoRV-mosaic plasmid control. Each assay could detect between 10^0^–10^7^ copies/reaction. Testing of the different KoRV subtype controls gave quantification cycle (Cq) assay values that averaged 20 for 10^7^ copies, 23 for 10^6^ copies, 26 for 10^5^ copies, 29 for 10^4^ copies, 32 for 10^3^ copies, 35 for 10^2^ copies, 39 for 10^1^ copies, and 42 for 10^0^ copies with an average correlation coefficient (R^2^) of 0.995 for the replicates. Each assay also showed 100% specificity for detection of the KoRV-specific (A, B, E, F and J) *env* sequences, except for the KoRV-B test which also detects KoRV-J. Nonetheless, the KoRV-J-specific assay did not detect KoRV-B *env* sequences and can be used to distinguish these two genotypes.

### Distribution of KoRV variants and VLs in wild and zoo koalas

Koalas were considered infected with a specific subtype if any specimen was positive for either KoRV-specific DNA or RNA sequences. For koalas with multiple blood specimens, a KoRV subtype was considered present if any specimen was positive for that subtype and the VL was averaged over the specimens. Koala β-actin sequences were detected at expected levels in all specimens with a mean and median of 6.39 and 6.47 log10 copies/ug PBMC DNA, 5.29 and 5.19 log10 copies/mL plasma RNA, 6.73 and 6.87 log10 copies/ug tissue DNA, and 5.53 and 5.66 log10 copies/ug tissue RNA, respectively. Table 2 provides a summary of the distribution of KoRV subtypes in koalas by institution location. The generic KoRV qPCR assay identified infection in specimens from all 126 koalas. Testing of PBMC and tissue DNA using the type-specific qPCR assays for detection of KoRV provirus found that all koalas in our study were infected with KoRV-A.

**Table 2:** Distribution of koala retrovirus (KoRV) subtypes in captive and wild koalas

Site (totals)	KoRV generic (%)	KoRV-A-specific (%)	KoRV-A only (%)	KoRV-B-specific (%)	KoRV-J-specific^a^ (%)	KoRV-F-specific (%)	KoRV-E-specific (%)
US zoos (n = 78)	78/78 (100)	78/78 (100)	70/78 (89.7)	9/78 (10.3)	1/78 (1.3)	/78 (7.7)	4/78 (5.1)
Australian zoos (n = 27)	27/27 (100)	27/27 (100)	11/27 (40.7)	15/27 (59.3)	3/27 (11.1)	0/27 (37.0)	11/27 (40.7)
St. Bees Island (n = 21)	21/21 (100)	21/21 (100)	21/21 (100)	0/21 (0)	ND	0/21 (0)	0/21 (0)
Total (n = 126)	126/126 (100)	126/126 (100)	102/126 (81.0)	24/126 (18.3)	4/126 (3.2)	16/126 (12.7)	15/126 (11.9)

Results include testing of any specimen type available for DNA and/or RNA KoRV sequences per koala. ND, not done

^a^KoRV-J testing was only done if a koala tested positive for KoRV-B to distinguish these two subtypes since they are very similar genetically

Most koalas in the 10 U.S. zoos were infected with KoRV-A only (70/78, 89.7%) (Table 2). A small number of koalas (10.3%) at the LA Zoo were infected with non-A subtypes. A wider diversity of KoRV was seen in 16/27 (59.3%) koalas at the Australian zoos and were co-infected with at least one additional non-KoRV-A genotype, compared to those at US zoos. Of 24 koalas infected with KoRV-B, four were KoRV-J-infected, 15 were KoRV-F-infected, and 14 had KoRV-E. The most common mixed subtype infection was KoRV-A, -B, -F, -E (n = 9). Two koalas were infected with subtypes A, B, J, E, and F, one was infected with KoRV-A, -B, -J, and -F, and three koalas each were infected with KoRV-A, -B, -E or KoRV-A, -B -F. One koala was infected with subtypes A, B, and J. In contrast, all 21 wild koalas from St. Bees Island were infected with only KoRV-A.

**Fig. 1:**
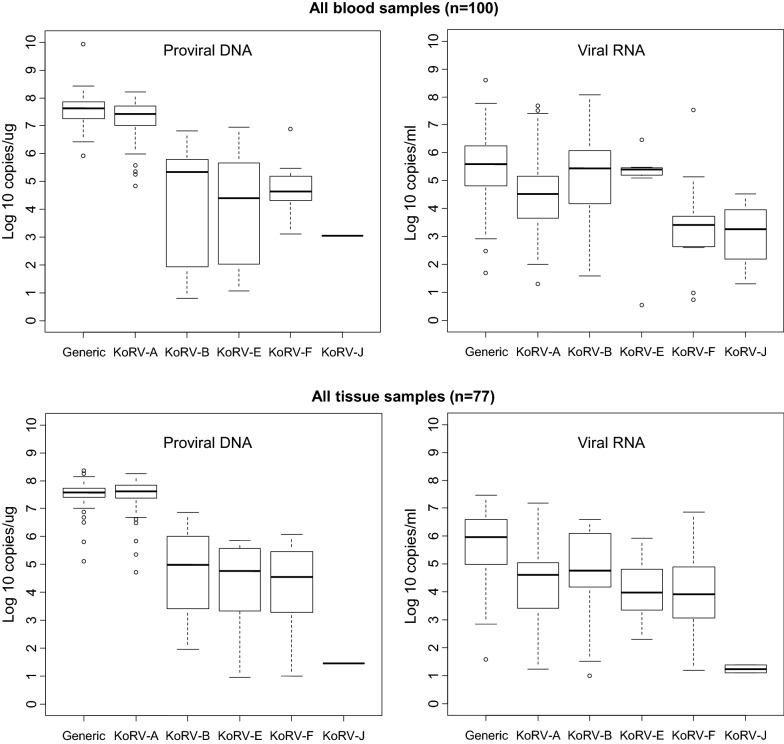
Distribution and quantity of koala retrovirus (KoRV) subtypes in all koala blood and tissue specimens. Box and whisker plots showing median and interquartile ranges of log10 KoRV levels in genomic DNA (copies/ug) from peripheral blood mononuclear cells and/or tissues obtained at necropsy on the left and the plasma (copies/mL) and/or tissue (copies/ug) RNA levels on the right in each panel. Numbers in parentheses indicate numbers of specimens tested and include multiple specimens from some koalas. For blood samples, viral loads from animals with multiple collection dates were averaged for a total of 100 koalas with blood specimens. Tissues were only available from koalas at US zoos and were not averaged if multiple tissues were from the same animal. Tissue specimens included liver, skin, spleen, lung, lymph node, brain, nodules, cerebrum, heart, kidney, bone marrow, thymus, skin, muscle, and intestine. The ends of the whiskers extend to the largest (smallest) value less than (greater than) or equal to 1.5 interquartile ranges above (below) the third (first) quartile, respectively. Observations beyond the whiskers are indicated by open circles. Samples with test results below the limit of detection were not included in the plots

**Fig. 2:**
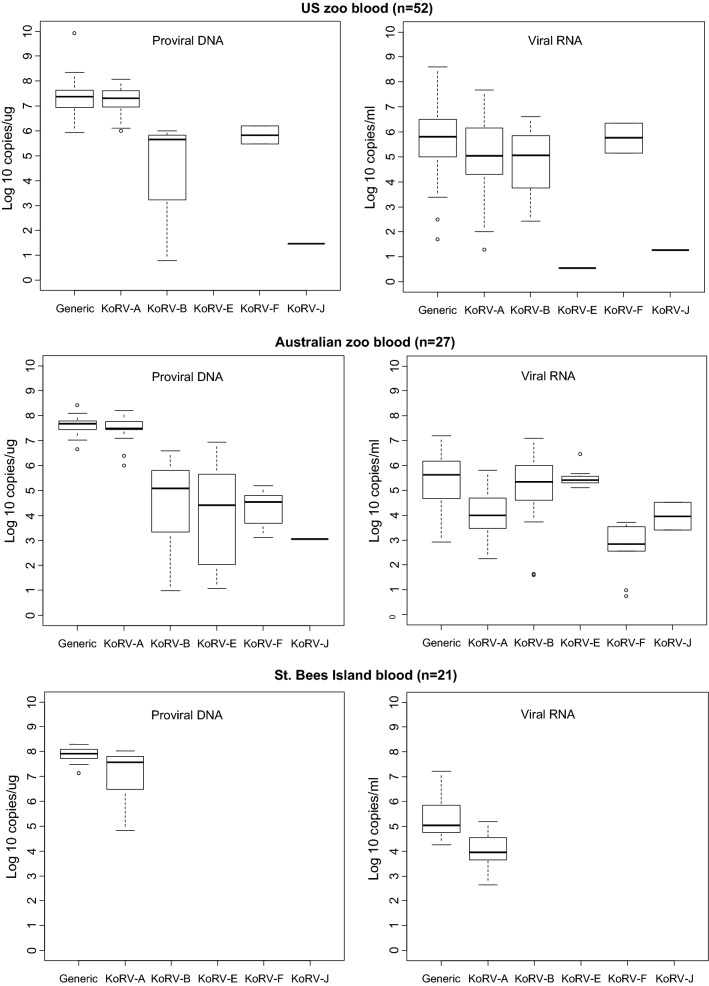
Distribution and quantity of koala retrovirus (KoRV) subtypes in koala blood by participating study sites. Box and whisker plots showing median and interquartile ranges of log10 KoRV levels in genomic DNA (copies/ug) from peripheral blood mononuclear cells on the left and the plasma (copies/mL) RNA levels on the right of each panel. Numbers in parentheses indicate number of koalas tested. The ends of the whiskers extend to the largest (smallest) value less than (greater than) or equal to 1.5 interquartile ranges above (below) the third (first) quartile, respectively. Observations beyond the whiskers are indicated by open circles. Samples with test results below the limit of detection were not included in the plots

**Fig. 3:**
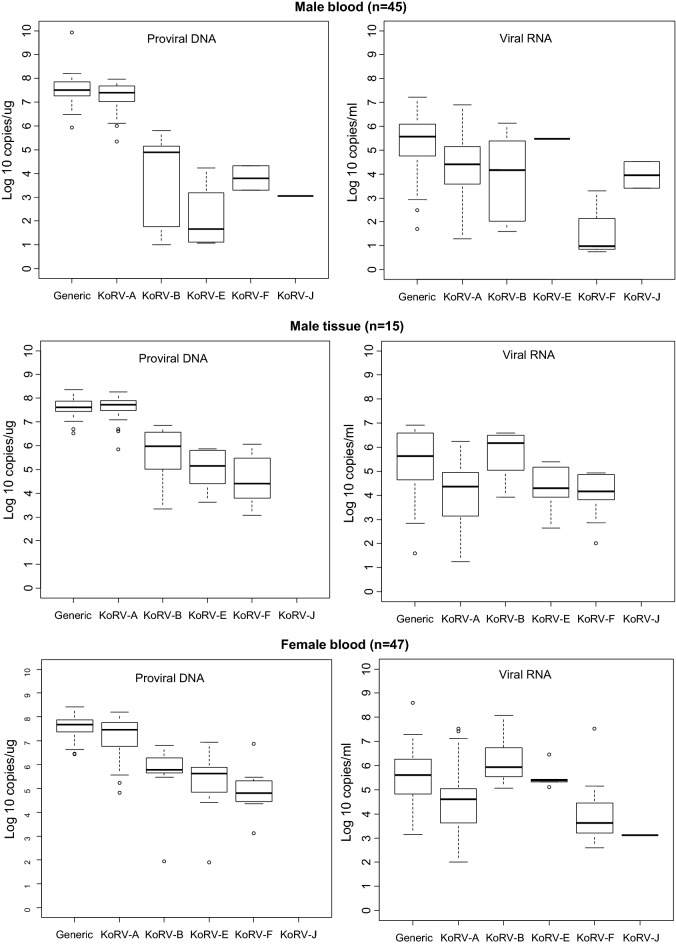

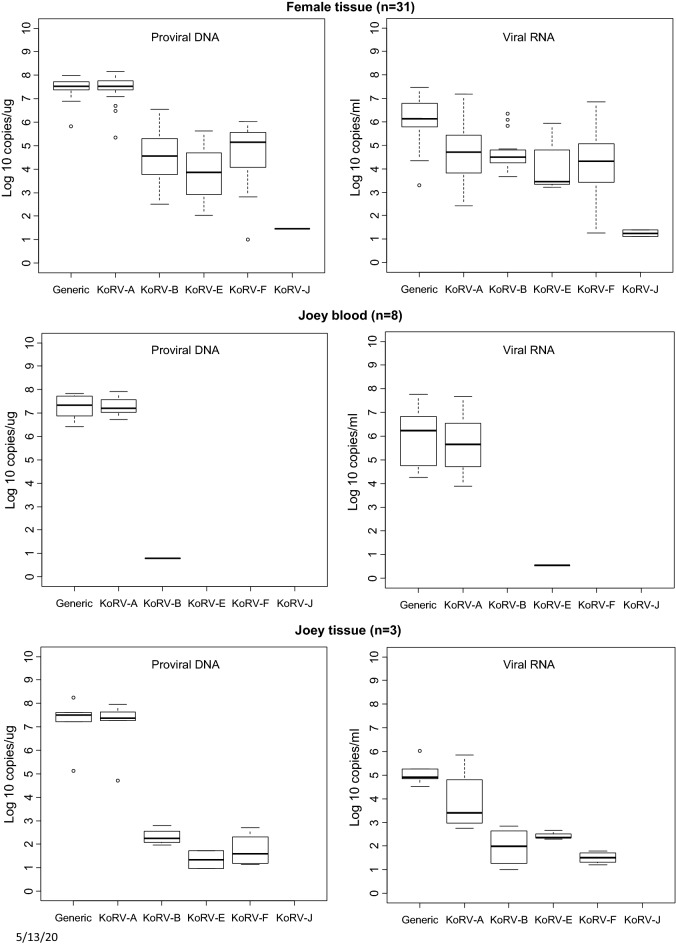
Distribution and quantity of koala retrovirus (KoRV) subtypes in male, female, and joey koalas. Box and whisker plots showing median and interquartile ranges of log10 KoRV levels in genomic DNA (copies/ug) from peripheral blood mononuclear cells and or tissues obtained at necropsy on the left and the plasma (copies/mL) and/or tissue (copies/ug) RNA levels on the right of each panel. Numbers in parentheses indicate number of koalas tested for blood and tissue samples. All tissues for each animal were included and multiple blood samples from each koala were averaged. The ends of the whiskers extend to the largest (smallest) value less than (greater than) or equal to 1.5 interquartile ranges above (below) the third (first) quartile, respectively. Observations beyond the whiskers are indicated by open circles. Samples with test results below the limit of detection were not included in the plots

**Fig. 4:**
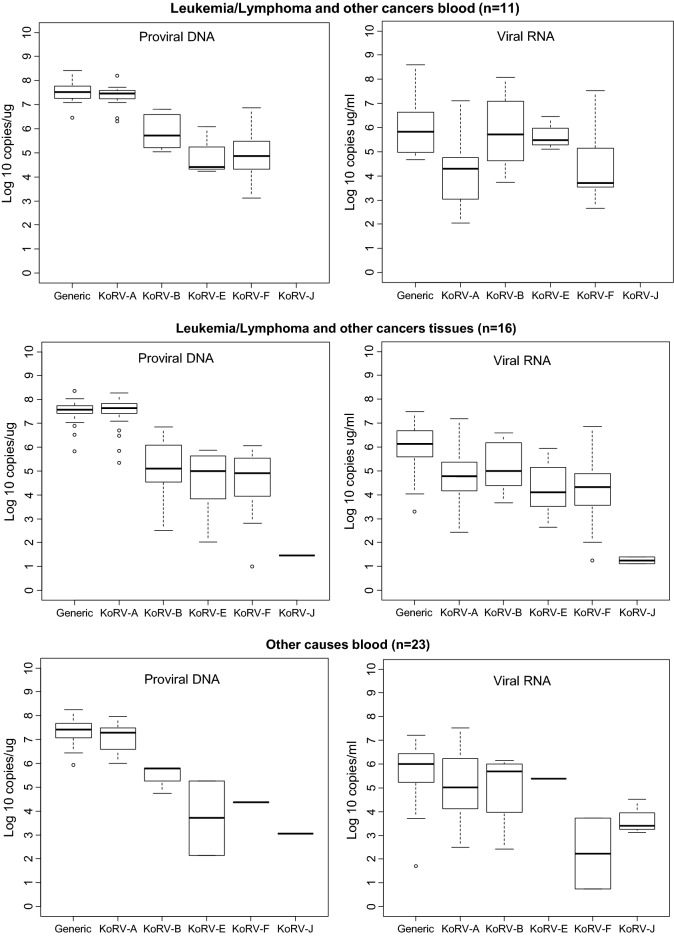

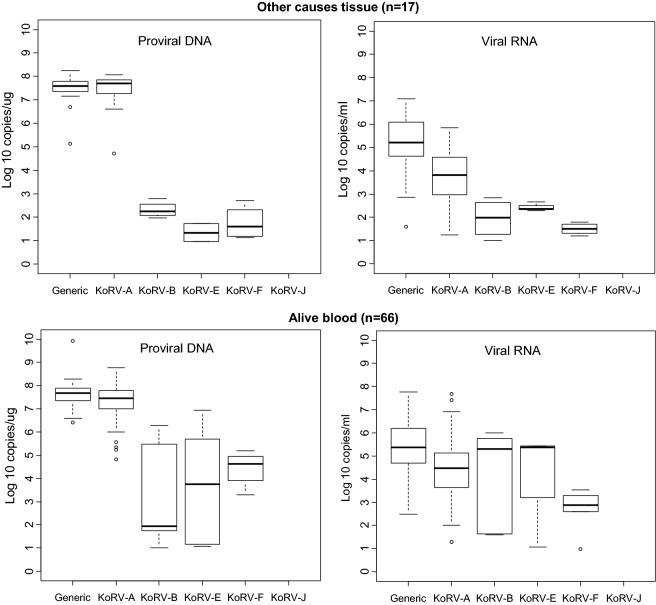
Distribution and quantity of koala retrovirus (KoRV) subtypes in koalas with and without disease. Box and whisker plots showing median and interquartile ranges of log10 KoRV levels in genomic DNA (copies/ug) from peripheral blood mononuclear cells and/or tissues obtained at necropsy on the left and the plasma (copies/mL) and/or tissue (copies/ug) RNA levels on the right of each panel. Numbers in parentheses indicate number of koalas tested for blood and tissue samples. All tissues for each animal were included and multiple blood samples from each koala were averaged. Other cancers include osteoma, metastatic sarcoma, osteosarcoma, hemangiosarcoma, and mammary carcinoma. Other causes of death include anemia, weight loss, old age, euthanasia, acute respiratory illness, torsion, intussusception, pulmonary interstitial fibrosis, intestinal volvulus, unknown, and early pouch young death. The ends of the whiskers extend to the largest (smallest) value less than (greater than) or equal to 1.5 interquartile ranges above (below) the third (first) quartile, respectively. Observations beyond the whiskers are indicated by open circles. Samples with test results below the limit of detection were not included in the plots

The distribution of VLs for each detected KoRV subtype is shown in the box plots in Figs. 1, 2, 3 and 4. Figure 1 shows the overall distribution in blood and tissue specimens, while Figs. 2, 3 and 4 show the distribution by institution location, gender, and health status, respectively. In each case, distributions are shown separately for each specimen type (blood and tissue). For blood samples, VLs from animals with multiple collection dates were averaged for a total of 100 koalas with blood specimens. Tissues were only available from koalas at US zoos and were not averaged when multiple tissues were from the same animal. Plasma and tissue RNA levels were not directly comparable since the copies per unit differ.

Although the KoRV-A median log10 pVLs (7.43) were higher for blood PBMCs than non-A subtypes (range 2.26–6.36) for all koala characteristics, the KoRV-A median log10 blood plasma VLs (4.62) were lower than the median log10 plasma VLs for KoRV-B, -E, and -F (6.26, 5.39, and 6.145, respectively) (Fig. 1). Similarly, the median plasma VLs were higher than KoRV-A for subtype F at US zoos (5.76 vs 5.04), subtypes B and E at Australian zoos (5.35 and 5.41 vs 4.00), subtype E for males (5.48 vs 4.41), and subtype B and E for females (5.93 and 5.40 vs 4.61) (Figs. 2, 3). Median subtype B and F plasma VLs were about two log10 copies higher for female than male koalas (5.93 and 3.63 vs 4.18 and 1.82, respectively) (Fig. 3). The median KoRV-B, -E, and -F plasma VLs (5.07, 5.48, and 5.15, respectively) were at least one log10 higher than those for KoRV-A (3.78) in koalas with leukemia/lymphoma (Fig. 4). Similarly, the median KoRV-B and KoRV-E plasma VLs (6.38 and 5.78, respectively) were at least one log10 higher than those for KoRV-A (4.53) in koalas with other cancers. For koalas that died from other causes or were alive the differences in the median plasma VLs were than less than one log10 for KoRV-B (5.70 or 5.30, respectively) and KoRV-E (5.39 or 5.37, respectively) compared to KoRV-A (5.01 or 4.48, respectively) (Fig. 4).

As for the koala blood DNA specimens, KoRV-A in general showed the highest median pVLs in tissue gDNA for all koala characteristics (Figs. 1, 2, 3 and 4). Unlike plasma samples, male tissues had almost twice the median gDNA levels for subtype B compared to females (7.17 vs 4.49, respectively) (Fig. 3). Subtype B gDNA pVLs were only slightly higher than those for KoRV-A (5.00 vs 4.91, respectively) (Fig. 4). KoRV-A was the only subtype found in tissues from koalas with other cancers (Fig. 4).

The generic KoRV qPCR test for total KoRV expression gave nearly equivalent median pVLs as for KoRV-A using the type specific assay with non-A subtypes having at least 1.5-fold less median pVLs. In contrast, plasma viral expression was greater for at least one of the non-A KoRVs compared to the KoRV-A specific assay, except for female tissues, tissues from koalas that died from other cancers, and joey specimens or when a koala was infected with only KoRV-A (Figs. 1, 2, 3 and 4).

### Association of KoRV subtypes with koala demographic and clinical variables

We next investigated potential associations between KoRV subtype, in both RNA and gDNA for tissue (Table 3) and blood samples (Table 4), and age in years, gender, and final health status classification. Associations between subtype and koala locations were only presented in Table 4 for blood samples, as tissue samples were available only for koalas at US zoos. The outcome variable was defined as infection with KoRV-B, -E, and -F for any specimen measurement of each individual koala. The *p* values were determined comparing the distributions of variables in koalas infected with each KoRV subtype separately and did not include KoRV-A since all koalas in our study population had the endogenous KoRV-A. KoRV subtypes B, E, and F were observed to have similar associations with age, sex and final health status classification in both RNA and DNA samples. For tissue samples, we observed that the positive koalas were significantly younger than the negative koalas in both RNA and DNA measurements (Table 3). However, for blood samples, of these four koala characteristics only the presence of health status classification and cause of death and animal location showed significance for subtypes B and F. For example, for koalas infected with subtype B by RNA testing, 37.5% (6/16) died from leukemia-lymphoma and other cancers, compared to 7.1% (6/84) of koalas infected with only KoRV-A. No significant age difference was found comparing the negative (KoRV-A-only) and positive (also KoRV-B infected) populations. The presence of the non-A KoRV subtypes in both RNA and DNA specimens was also significantly different by koala location for blood samples (*p* < 0.0001, Table 4) with the majority of subtype-B-positive RNA (81.3%, 13/16) and subtype-B-positive DNA (83.3%, 15/18) koalas being from the Australian zoos (Table 4).

**Table 3:** Association between KoRV RNA and DNA subtype detection and selected koala characteristics by subject for tissue samples

Characteristic	KoRV-B			KoRV-E			KoRV-F		
	Positive (N = 5)	Negative (N = 26)	*p* value	Positive (N = 3)	Negative (N = 28)	*p* value	Positive (N = 5)	Negative (N = 26)	*p* value
RNA									
Age (years)	3.3	9.5	0.04	2.7	9.3	0.05	3.3	9.5	0.04
Sex			0.04			0.3			0.04
Female	2 (40.3%)	11 (42.3%)		1 (33.3%)	12 (42.9%)		2 (40.0%)	11 (42.3%)	
Male	1 (20.0%)	14 (53.9%)		1 (33.3%)	14 (50.0%)		1 (20.0%)	14 (53.9%)	
Joey	2 (40.3%)	1 (3.9%)		1 (33.3%)	2 (7.1%)		2 (40.0%)	1 (3.9%)	
Health status classification and cause of death^a^			0.7			0.6			0.7
Leukemia-lymphoma and other cancer	3 (60.0%)	13 (50.0%)		2 (66.7%)	14 (50.0%)		3 (60.0%)	13 (50.0%)	
Other	2 (40.0%)	13 (50.0%)		1 (33.3%)	14 (50.0%)		2 (40.0%)	13 (50.0%)	

Characteristic	Positive (N = 6)	Negative (N = 25)	*p* value	Positive (N = 3)	Negative (N = 28)	*p* value	Positive (N = 5)	Negative (N = 26)	*p* value
DNA									
Age (years)	4	9.6	0.04	2.7	9.3	0.05	3.3	9.5	0.04
Sex			0.1			0.3			0.04
Female	2 (33.3%)	11 (44.0%)		1 (33.3%)	12 (42.9%)		2 (40.0%)	11 (42.3%)	
Male	2 (33.3%)	13 (52.0%)		1 (33.3%)	14 (50.0%)		1 (20.0%)	14 (53.9%)	
Joey	2 (33.3%)	1 (4.0%)		1 (33.3%)	2 (7.1%)		2 (40.0%)	1 (3.9%)	
Health status classification and cause of death^a^			0.4			0.6			0.7
Leukemia-lymphoma and other cancer	4 (66.7%)	12 (48.0%)		2 (66.7%)	14 (50.0%)		3 (60.0%)	13 (50.0%)	
Other	2 (33.3%)	13 (52.0%)		1 (33.3%)	14 (50.0%)		2 (40.0%)	13 (50.0%)	

KoRV was considered detected if any sample at any time point from a koala tested positive for that specific subtype

^a^Causes of death at study conclusion are listed for other than alive. Other cancers included osteoma, metastatic sarcoma, osteosarcoma, hemangiosarcoma, and mammary carcinoma. Other causes of death included anemia, old age, euthanasia, acute respiratory illness, intestinal volvulus, torsion, intussusception, pulmonary interstitial fibrosis, unknown, and early young pouch death. The most common lesion in koalas that were classified with death at old age was degenerative joint disease

**Table 4:** Association between KoRV RNA and DNA subtype detection and selected koala characteristics by subject for blood samples

Characteristic	KoRV-B			KoRV-E			KoRV-F		
	Positive (N = 16)	Negative (N = 84)	*p* value	Positive (N = 8)	Negative (N = 92)	*p* value	Positive (N = 12)	Negative (N = 88)	*p* value
RNA									
Age (years)	5.5	5.8	0.8	4.4	5.9	0.4	4.8	5.9	0.4
Sex			0.4			0.2			0.3
Female	8 (50.0%)	39 (46.4%)		6 (75.0%)	41 (44.6%)		8 (66.7%)	39 (44.3%)	
Male	8 (50.5%)	37 (44.0%)		1 (12.5%)	44 (47.8%)		4 (33.3%)	41 (46.6%)	
Joey	0 (0.0%)	8 (9.5%)		1 (12.5%)	7 (7.6%)		0 (0.0%)	8 (9.1%)	
Health status classification and cause of death^a^			0.002			0.07			0.003
Alive	6 (37.5%)	60 (71.4%)		4 (50.0%)	62 (67.4%)		5 (41.7%)	61 (69.3%)	
Leukemia-lymphoma and other cancer	6 (37.5%)	6 (7.1%)		3 (37.5%)	9 (9.8%)		5 (41.7%)	7 (8.0%)	
Other	4 (25.0%)	18 (21.4%)		1 (12.5%)	21 (22.8%)		2 (16.7%)	20 (22.7%)	
Location			< 0.0001			0.0003			< 0.0001
Australian zoos	13 (81.3%)	14 (16.7%)		7 (87.5%)	20 (21.7%)		10 (83.3%)	17 (19.3%)	
US zoos	3 (18.8%)	49 (58.3%)		1 (12.5%)	51 (55.4%)		2 (16.7%)	50 (56.8%)	
Wild	0 (0.0%)	21 (25.0%)		0 (0.0%)	21 (22.8%)		0 (0.0%)	21 (23.9%)	

Characteristic	Positive (N = 18)	Negative (N = 82)	*p* value	Positive (N = 11)	Negative (N = 89)	*p* value	Positive (N = 10)	Negative (N = 90)	*p* value
DNA									
Age (years)	4.9	6	0.3	5.7	5.8	1	4.5	5.9	0.4
Sex			0.9			0.4			0.08
Female	9 (50.0%)	38 (46.3%)		7 (63.6%)	40 (44.9%)		8 (80.0%)	39 (43.3%)	
Male	8 (44.4%)	37 (45.1%)		4 (36.4%)	41 (46.1%)		2 (20.0%)	43 (47.8%)	
Joey	1 (5.6%)	7 (8.5%)		0 (0.0%)	8 (9.0%)		0 (0.0%)	8 (8.9%)	
Health status classification and cause of death^a^			0.009			0.3			0.0005
Alive	9 (50.0%)	57 (69.5%)		6 (54.6%)	60 (67.4%)		4 (40.0%)	62 (68.9%)	
Leukemia-lymphoma and other cancer	6 (33.3%)	6 (7.3%)		3 (27.3%)	9 (10.1%)		5 (50.0%)	7 (7.8%)	
Other	3 (16.7%)	19 (23.2%)		2 (18.2%)	20 (22.5%)		1 (10.0%)	21 (23.3%)	
Location			< 0.0001			< 0.0001			0.0003
Australian zoos	15 (83.3%)	12 (14.6%)		11 (100.0%)	16 (18.0%)		8 (80.0%)	19 (21.1%)	
US zoos	3 (16.7%)	49 (59.8%)		0 (0.0%)	52 (58.4%)		2 (20.0%)	50 (55.6%)	
St. Bees Island (wild)	0 (0.0%)	21 (25.6%)		0 (0.0%)	21 (23.6%)		0 (0.0%)	21 (23.3%)	

KoRV was considered detected if any sample at any time point from a koala tested positive for that specific subtype

^a^Causes of death at study conclusion are listed for other than alive. Other cancers included osteoma, metastatic sarcoma, osteosarcoma, hemangiosarcoma, and mammary carcinoma. Other causes of death included anemia, old age, euthanasia, acute respiratory illness, intestinal volvulus, torsion, intussusception, pulmonary interstitial fibrosis, unknown, and early young pouch death. The most common lesion in koalas that were classified with death at old age was degenerative joint disease

### Model-based analysis of KoRV subtype VLs and koala demographic and clinical characteristics

We then combined all available KoRV plasma RNA and gDNA VL blood-sample results, except for subtype J which was only detected in a few animals, to explore potential associations between VL and the available koala characteristics (Table 5). In this analysis, we included all specimen measurements, including those that were not detectable (below the assay detection limit, BLD). Results are not reported for tissue samples as the Tobit model did not converge in that analysis. For the blood sample analysis, we included subtype comparisons with subtype B as the reference and compared the variables age at testing, sex, final health status classification, and animal location. Koala age and sex were not significantly associated with VL levels after controlling for other variables.

**Table 5:** Comparisons of differences in KoRV plasma RNA and blood genomic DNA (gDNA) log10 viral loads (VLs) and koala characteristics in blood samples

Variables	Plasma mean difference	Plasma 95% lower limit	Plasma 95% upper limit	Plasma *p* value	gDNA mean difference	gDNA 95% lower limit	gDNA	gDNA *p* value
							95% upper limit	
Subtype				< 0.0001				< 0.0001
KoRV-A vs. KoRV-B	4.47	3.71	5.22	< 0.0001	7.13	6.41	7.85	< 0.0001
KoRV-E vs. KoRV-B	− 2.8	− 3.79	− 1.81	< 0.0001	− 2.48	− 3.4	1.57	< 0.0001
KoRV-F vs. KoRV-B	2.12	0.93	3.32	0.0007	− 0.71	− 1.5	0.07	0.07
Age at Testing	− 0.03	− 0.14	0.08	0.6	− 0.02	− 0.12	0.08	0.6
Sex				0.2				0.3
Male vs. Female	− 0.67	− 1.47	0.13	0.1	− 0.6	− 1.34	0.15	0.3
Joey vs. Female	0.1	− 1.66	1.86	0.9	− 0.62	− 2.3	1.06	0.8
Joey vs. Male	0.74	− 1.00	2.47	0.4	− 0.02	− 1.67	1.63	1
Health status classification and cause of death^a^				0.002				0.0005
Alive vs. Other	− 0.55	− 1.74	0.65	0.4	0.17	− 0.92	1.27	0.8
Leukemia-lymphoma or other cancers vs. Other	1.47	0.14	2.81	0.03	2.18	0.93	3.42	0.0008
Leukemia-lymphoma or other cancers vs. Alive	2.01	0.89	3.13	0.0006	2.01	0.94	3.07	0.0003
Location				0.004				< 0.0001
Australian zoos vs. Wild	1.87	0.59	3.15	0.002	2.67	1.49	3.84	< 0.0001
US zoos vs. Wild	0.55	− 0.66	1.75	0.4	0.26	− 0.84	1.37	0.6
Australian zoos vs. US zoos	1.31	0.42	2.19	0.004	2.40	1.57	3.24	< 0.0001

All VL measurements, including undetectable measurements, from each koala were used in the Tobit model, including multiple samples collected at different time points or multiple samples collected from the same time point, i.e. at necropsy

^a^Causes of death at study conclusion are listed for other than alive. Other cancers included osteoma, metastatic sarcoma, osteosarcoma, hemangiosarcoma, and mammary carcinoma. Other causes of death included anemia, old age, euthanasia, acute respiratory illness, intestinal volvulus, torsion, intussusception, pulmonary interstitial fibrosis, unknown, and early young pouch death. The most common lesion in koalas that were classified with death at old age was degenerative joint disease

Overall, we found that different subtypes showed significantly different VLs compared to subtype B. For example, for plasma RNA the mean log10 VLs for KoRV-A and KoRV-F were significantly higher than that for KoRV-B (mean difference = 4.47, 95% CI 3.71, 5.22, *p* < 0.0001 and mean difference = 2.12, 95% CI 0.93, 3.32, *p* = 0.0007, respectively). The mean log10 VLs for KoRV-E was significantly lower than that for subtype B (mean difference = − 2.80, 95% CI − 3.79, − 1.81, *p* < 0.0001). Compared to other causes, koalas that died from leukemia-lymphoma or other cancers had significantly higher VLs compared to those that died from other causes (mean difference = 1.47, 95% CI 0.14, 2.81, *p* = 0.03) and compared to those alive (mean difference = 2.01, 95% CI 0.89, 3.13, *p* = 0.0006).

When controlling for other covariates including subtype, koalas at the Australian zoos were found to have the highest mean VLs. When compared to wild koalas the mean difference was 1.87 (95% CI 0.59, 3.15), while compared to koalas in US zoos the mean difference was 1.31 (95% CI 0.42, 2.19). Analysis of the pVLs gave results similar to the plasma RNA VLs with koalas with leukemia-lymphoma or other cancers again having significantly higher pVLs compared to those that died from other causes (mean difference = 2.18, 95% CI 0.93, 3.42, *p* = 0.0008) or that were alive (mean difference = 2.01, 95% CI 0.94, 3.07, *p* = 0.0003).

### Longitudinal KoRV blood levels, diversity, and antiretroviral treatment

Longitudinal blood specimens were available for eight koalas (three males, four females, one joey) at six zoos. Of these eight, five adult koalas were infected with only KoRV-A, which showed fluctuating KoRV levels over time. For example, plasma VLs decreased 0.8 and 1.2 logs over 8 months and about 0.4 logs over 2 years for two KoRV-A-infected male koalas at US zoos. In contrast, plasma KoRV-A VLs increased 0.7 logs over 19 days for a female at the Taronga Zoo. The one joey was infected with low levels of KoRV-B in the PBMCs and KoRV-E in the plasma at 9 months of age but both KoRV-B and -E were undetectable 19 days later. Two adults with longitudinal specimens died from leukemia/lymphoma. The male housed at the Taronga Zoo, had two specimens collected 19 days apart and was infected with KoRV-A, -B, -F and –E at both time points and had DNA and RNA levels within 0.5 logs, except for KoRV-E DNA levels that increased by almost 2.5 logs at the later time point. The female (named Brooklyn) housed at the LA Zoo had 12 samples collected every 3–4 days over a month. KoRV-A, -B, -F were detected at each time point, while KoRV-J was detected intermittently with 3/12 plasma RNA specimens testing positive while all matching PBMC DNA specimens were KoRV-J-negative.

Brooklyn received antiretroviral treatment with integrase (raltegravir) and nucleotide reverse transcriptase (tenofovir) inhibitors for 33 days for experimental treatment of her leukemia/lymphoma. While KoRV-A levels in Brooklyn were relatively stable over time averaging 7.10 and 7.59 log10 copies for plasma and PBMCs, respectively, the KoRV-B and -F averages were 1.24 (8.05 vs 6.81) and 0.67 (7.54 vs 6.87) logs higher in plasma than in PBMC DNA (Additional file 1: Fig. S1), respectively. Mean KoRV-B and -F plasma VLs were also 0.95 and 0.44 logs higher, respectively, than KoRV-A plasma levels. There was about a one log decrease in all KoRV plasma levels except for KoRV-F at day 7 post treatment (pt) that rebounded to pre-treatment levels four days later followed by another drop in plasma VLs at 21–28 days pt and for KoRV-A at 33 days pt. KoRV-J plasma levels were low with a mean of 1.3 log10 copies/ml when detected. After not responding to treatment and continued poor health, she was euthanized on day 33 pt.

### Transmission of KoRV subtypes and associated diseases

To investigate possible transmission patterns of KoRV variants and disease associations in koalas we mapped detection of each subtype in offspring and their recorded parents when those specimens were available. From animal records we identified 66 offspring with KoRV test results for both parents (n = 40) or from the dam (n = 15) or sire (n = 11) only. To evaluate dam-to-offspring transmission we examined the KoRV profiles and disease status of 55 offspring and their dams (Table 6). The age at testing of the 55 offspring ranged from 1 to 262 months with a mean and median of 53.7 and 40 months, respectively.

**Table 6:** Non KoRV-A subtype profiles and health status in 55 dam and offspring pairs

Dam name	Dam age at testing (mos)	Dam KoRV profile^a^	Dam Health Status Classification^b^	Offspring Name	Offspring sex	Offspring age at testing (mos)	Offspring Health Status Classification^b^	Offspring KoRV profile^a^
Amaroo	171	−−−−	Alive	Coombah	M	105	Old age	−−−−
Amaroo	171	−−−−	Alive	Cynthia	F	27	Alive	−−−−
Amaroo	171	−−−−	Alive	Maloo	M	132	Old age	−−−−
Colliet	182	−−−−	Old age	Kookoora	M	23	Alive	−−−−
Colliet	182	−−−−	Old age	Nyoonbi	M	28	Alive	−−−−
Colliet	182	−−−−	Old age	Wonnewarra	F	118	Old age	−−−−
Coombah	105	−−−−	Old age	Cambee	J	12	Alive	−−−−
Georgie	NA	−−−−	Leukemia-lymphoma	Jimmy	M	72	Alive	−−−−
Georgie	NA	−−−−	Leukemia-lymphoma	Karri	F	73	Alive	−−−−
Karri	71	−−−−	Alive	Ceduna	F	13	Alive	−−−−
Karri	71	−−−−	Alive	Kathrine	J	10	Alive	−−−−
Karri	71	−−−−	Alive	Logan	J	12	Alive	−−−−
Karri	71	−−−−	Alive	MacKenzie	J	9	Alive	+−+
Kathrine	10	−−−−	Alive	Edmund	J	9	Alive	−−−−
Kemba	71	−−−−	Anemia	Killara	F	98	Leukemia-lymphoma	−−−−
Lottie	126	−−−−	Alive	Oliver	M	44	Alive	−−−−
Lottie	126	−−−−	Alive	Owyn	F	34	Leukemia-lymphoma	−−−−
Lottie	126	−−−−	Alive	Oz	M	64	Alive	−−−−
Lou	84	−−−−	Other causes	Irwin	M	57	Alive	+−−−
Lou	84	−−−−	Other causes	Seeana	F	22	Alive	−−−−
Maggie	115	−−−−	Old age	Freya	F	61	Alive	+−++
Maggie	115	−−−−	Old age	Lincoln	M	45	Alive	−−−−
Maggie	115	−−−−	Old age	McAuley	M	99	Other causes	++−−
Maggie	115	−−−−	Old age	Rubi	F	26	Alive	−−−−
Maggie	115	−−−−	Old age	Willow	J	12	Alive	−−−−
Midgee	183	−−−−	Other cancers	Austin	M	26	Alive	−−−−
Midgee	183	−−−−	Other cancers	Wanneroo	F	19	Alive	−−−−
Midgee	183	−−−−	Other cancers	Wruwallin	F	114	Alive	−−−−
Minnie	59	−−−−	Leukemia-lymphoma	Sheila	J	7	Anemia	−−−−
Orana	173	−−−−	Other cancers	Colliet	F	182	Old age	−−−−
Orana	173	−−−−	Other cancers	Kobi	M	75	Other cancers	−−−−
Orana	173	−−−−	Other cancers	Miah	F	23	Anemia	−−−−
Orana	173	−−−−	Other cancers	Mundooie	M	128	Old age	−−−−
Orana	173	−−−−	Other cancers	Sooky	F	29	Alive	−−−−
Owyn	22	−−−−	Leukemia-lymphoma	Kennedy	J	12	Alive	−−−−
Owyn	22	−−−−	Leukemia-lymphoma	Quincy	J	10	Alive	−−−−
Tilly	38	−−−−	Other causes	Wanda	F	60	Alive	−−−−
Wanda	60	−−−−	Alive	River	M	25	Alive	+−+−
Wanda	60	−−−−	Alive	Tilly	F	38	Other causes	−−−−
Wruwallin	114	−−−−	Alive	Bunyip	M	262	Old age	−−−−
Wruwallin	114	−−−−	Alive	Kiki	F	60	Old age	−−−−
Carrie	142	+−− +	Alive	Erna	F	72	Other cancers	+−++
Carrie	142	+−− +	Alive	Grace	F	44	Alive	−−−−
Carrie	142	+−− +	Alive	Lou	F	85	Other causes	−−−−
Carrie	142	+−− +	Alive	Pepper	M	14	Alive	−−−−
Brooklyn	65	+−++	Leukemia-lymphoma	Abby	F	40	Leukemia-lymphoma	+−+−
Brooklyn	40	+−−+	Leukemia-lymphoma	No name	J	1	Miscarriage	+−+−
Erna	72	+−++	Other cancers	Lillian	F	39	Alive	+−−++
Freya	61	+−++	Alive	Ash	M	24	Leukemia-lymphoma	+−+−
Jane	47	+−++	Leukemia-lymphoma	Brooklyn	F	65	Leukemia-lymphoma	+ ++−
Jane	47	+−++	Leukemia-lymphoma	Parker	M	82	Leukemia-lymphoma	+−++
Netty	NA	+? ? ?	Other cancers	No Name	J	4	Miscarriage	+−++
Yindi	154	++++	Old age	Coral	F	56	Alive	+−++
Yindi	154	++++	Old age	Eliza	F	47	Other cancers	+−+ +
Yindi	154	++++	Old age	Jane	F	107	Leukemia-lymphoma	+−++

*M* male, *F* female, *J* joey

^a^ − and + indicate absence (below the level of detection) or presence of KoRV subtypes B, J, F, E, respectively. ?, indicates not enough specimen to test for that subtype. All koalas were infected with KoRV-A

^b^Causes of death at study conclusion are listed for other than alive. Other cancers included osteoma, metastatic sarcoma, osteosarcoma, hemangiosarcoma, and mammary carcinoma. Other causes of death included anemia, old age, euthanasia, acute respiratory illness, intestinal volvulus, torsion, intussusception, pulmonary interstitial fibrosis, unknown, and early young pouch death

We found within the 55 complete pairs of dam and offspring data, that 36 pairs were negative for subtype B. For three pairs, the dam was infected with subtype B but not the offspring and for five offspring with subtype B infection, their dam was only infected with KoRV-A. For 11 dam-offspring pairs, both koalas were infected with at least subtype B. Overall, we found that the dam KoRV subtype was significantly associated with the offspring subtypes (*p* < 0.0001). Within the group of dams infected with subtype B and other non-A subtypes, 11/14 (78.6%) of the offspring were also infected with subtype B and other non-A subtypes. Within the subtype B-negative dams, 5/41 (12.2%) of the offspring were at least subtype B-positive. These results indicate that dams with KoRV-B alone or with other non-A subtypes were more likely to transmit their non-A KoRV subtypes to their offspring, but that discordant transmission profiles exist.

We identified eight offspring (7 adults, 1 joey) with discordant KoRV infections from the dam, including one KoRV-A, -B and -E-infected dam that birthed three KoRV-A-only infected offspring (two females, one male). The sire of two of these offspring tested KoRV-A-positive only. One female offspring with KoRV-B, -F, and -E tested at 72 months was born to a dam with only KoRV-A; the sire’s name was not recorded. Of the four other offspring (three males, one joey) with discordant KoRV infections, the dams were all KoRV-A-positive only while the one male sire of three of these was infected with KoRV-B and other non-A variants. The sire’s name for the fourth offspring was not recorded.

We also evaluated if subtype transmission was associated with disease in the offspring. Of the 55 dam/offspring pairs, neoplasia was identified in 10 (18.2%) pairs with leukemia/lymphoma in 7 pairs and other cancers in three (Table 6). Of these 10 pairs, three were infected with KoRV-A only including two with leukemia/lymphoma and one with another cancer. Interestingly, of these 10 dam/offspring pairs, two sires (1 KoRV-B-infected, 1 KoRV-A-infected only) of four offspring had leukemia/lymphoma and for one dam/offspring pair with other cancers the sire had leukemia/lymphoma. Discordant results were observed for 15 dam/offspring pairs (27.3%) with four dams giving birth to six offspring having leukemia/lymphoma and four dams giving birth to 9 offspring having other cancers while all 15 offspring were neoplasia-free at time of sampling. Of these 8 dams, five were KoRV-A-infected only and three were also infected with KoRV-B. We found that the odds of death from leukemia/lymphoma or other cancers for the offspring was higher in the multi-infection group compared to the KoRV-A only infection group (OR = 9.87, 95% CI 1.63, 59.74, *p* = 0.01) though the 95% CI is large.

## Discussion

We developed highly sensitive and specific qPCR assays to further characterize KoRV molecular epidemiology, disease potential, and transmissibility in many koalas from the US and Australia. We show that non-A KoRV subtypes are present in zoo koalas in the US and Australia but were absent from all 21 wild koalas from St. Bees Island. The prevalence and levels of non-A subtypes in both plasma RNA and PBMC DNA was significantly higher in koalas at Australian zoos than in those at US zoos. Our results indicate that unlike KoRV-A, non-A KoRV subtypes are likely replication active, evolving exogenous retroviruses that are not yet widely distributed. These results are supported by analysis of integrated KoRV-A, -B, -D and -E in a wild koala using long-read genome sequence assembly, the greater genetic KoRV envelope diversity seen in non-A subtypes and by recent KoRV variant prevalence studies of wild koala populations [10, 14–16, 19, 20]. While the identification of novel Env variants is important for understanding the epidemiology and evolutionary history of KoRV, the presence of a complete, intact genome is required to confirm a specific KoRV variant is replication competent, as both endogenous and exogenous retroviruses can be defective. For example, others found defective KoRV-B sequences in both zoo and wild koalas [7] and subtypes D and E were shown to be defective viruses [16, 20, 21, 34].

Our findings also suggest that the breeding and animal management programs at each international zoo likely reflects the current level of local KoRV diversity. For example, following the original importation from Australia the majority of the initial US koala population were born at the San Diego Zoo, which in our study were infected by only KoRV-A and perhaps by chance, a founder effect, or animal management strategies, have not become infected with non-A KoRVs. All non-A KoRVs in the US were found in koalas that were housed at or originated from the LA Zoo where KoRV-B was first identified [22]. Some koalas at the LA Zoo originated from northeastern Australia where non-A KoRV subtypes were found in wild animals suggesting a likely introduction source of the non-A subtypes into US zoo koalas [10, 14–16]. In contrast, over half of the koalas at the Taronga Zoo in Australia are infected with KoRV-B and other non-A subtypes likely originating from their predominantly wild-born ancestors. These hypotheses are supported by recent studies reporting KoRV-B molecular prevalence of about 25–48% in wild koalas from Queensland [10, 14, 16] and the inferred evolutionary history of these divergent KoRVs [10, 16]. Koalas introduced onto St. Bees island were likely only infected with KoRV-A when they were re-located there in the 1930s from Proserpine, Queensland, which is about 130 km northeast of St. Bees Island on the mainland [37]. Interestingly, the koalas on St. Bees Island, and other island koala populations, have lower levels of genetic diversity than those from Southeast Queensland where they originated [37]. The St. Bees Island koalas also had less of the recently identified recombinant KoRVs (RecKoRV1) loci that contain endogenous retroelements [21]. Combined, these results indicate that geographical isolation likely kept the St. Bees Island koalas free from infection with non-A KoRVs or that they and the RecKoRV1 variants were lost to selection or genetic drift [21]. Importantly, the isolation of koalas on St. Bees Island and on other Australian Islands likely influences both koala and KoRV evolution and may not reflect that of wild koala populations on the mainland.

We did not observe a significant difference in the distribution and tissue levels of KoRV subtypes by gender or age for both RNA and DNA detection and levels similar to those reported for a large population of wild koalas infected with KoRV-A and -B [14]. In contrast, others reported finding KoRV-B infection in only nine of 25 females in 36 wild animals, but infection was not associated with age [31]. We also found a higher prevalence of KoRV-B-infected koalas at the Australian zoos (59%) than previously reported in wild koalas (< 48%) [10, 14, 16], which could be explained by increased transmission occurring in animals housed in close contact at zoos. In another study [10], 18 sick (most with inflammatory disease), wild koalas around Brisbane were mostly KoRV-B-infected (77.7%) and mostly male, though the study was limited by testing of small numbers of sick animals presented to veterinarians in the area. All four female koalas in that study were KoRV-D-infected and two were KoRV-B-infected. These 18 koalas were all adults, but ages were not estimated to evaluate an association of age with KoRV diversity.

The first study that identified KoRV-B reported finding an association of lymphoma with infection though this determination was based on small numbers of koalas [22]. To further evaluate this observation, we conducted statistical analyses to determine potential associations of infection with different KoRV subtypes with disease in our larger study population. For blood specimens, we found significantly higher proportions of leukemia/lymphoma and other cancers in koalas infected with KoRV-B, -E and -F than those infected with only KoRV-A, including finding more neoplasia in offspring infected with non-A subtypes. Our findings are consistent with those reported recently that found a significant association of KoRV-B infection in wild koalas with other neoplasias, including osteochondroma, mesothelioma, and a non-specified proliferative bone marrow condition [14]. However, a new study using next generation sequencing (NGS) identified subtypes A, B, D, I and G in wild koalas but did not find an association of subtype and neoplasia [16] but which contrasts to that from Fabijan *et. al* who reported higher pVLs and plasma VLs in koalas with neoplasia compared to other disease categories in wild koalas by using a generic KoRV polymerase qPCR assay [32]. These discrepancies could be from the different populations studied and methods used but highlight the importance of additional studies to evaluate disease associations of KoRV infection. For example, we were unable to determine if the presence of a single non-A subtype was associated with leukemia/lymphoma since co-infection with various combinations of non-A subtypes was common. Nonetheless, even animals infected with only KoRV-A in our study were susceptible to neoplasia, which has been reported in both wild and zoo koalas [14, 30].

Since retroviral disease can be associated with increased VLs, we also examined KoRV subtype levels in animals with disease by using the Tobit model, which included all detectable measurements and those that were BLD providing a more robust analysis of the data compared to an analysis of just the detectable VLs like those shown in Figs. 1, 2, 3 and 4. Overall, koalas with leukemia/lymphoma or other cancers had significantly higher VLs in both plasma and proviral DNA compared to those that were alive or those that died from other causes. A new report also showed that koalas with neoplasia had significantly higher pVLs and plasma VLs than other disease categories but KoRV subtyping was not reported [32], similar to the results of Tarlinton et al*.* [30]. Quigley et al*.* reported a significant association of KoRV-B infection and both chlamydial disease and neoplasia but then reported higher KoRV-D levels in healthy koalas [14, 15]. However, measurement of VLs in these studies was complicated using total nucleic acids isolated from plasma samples which can inflate total VLs coming from both cellular and cell-free compartments. Interestingly, based on the detectable measurements in our study, all non-A KoRV subtypes except J had higher median plasma VLs than KoRV-A even though KoRV-A as an endogenous retrovirus is expressed constitutively.

Limited data exist on the longitudinal follow-up of KoRV infections. We were able to measure VLs in eight koalas with longitudinal specimens, including two adults with leukemia/lymphoma. We found that proviral load and/or plasma VLs fluctuated over time by as much as 2.5-fold and even the disappearance of non-A subtypes in as little as 19 days. Our results are comparable to those of Quigley et al*.* who followed a cohort of 16 adult koalas for a period of 4 years with sampling about every 6 months and found variable KoRV-A, -B, -D, and -F expression profiles over time [15]. In 2005, Tarlinton et al*.* reported variations of plasma VLs in ten koalas and observed an increase in VLs over 18 months as animals progressed to neoplasia, but subtyping was not conducted then [30]. The longitudinal samples from the two koalas with leukemia/lymphoma in our study were collected after they were already diagnosed with cancer but their VL still fluctuated between 0.4–2.5 logs, especially for KoRV-E in the male koala. Combined, these findings suggest that KoRV subtype replication is active and dynamic and may be driven by factors affecting host biological systems, including co-infection.

Although chlamydial infection was not present in our study population, it remains to be determined whether a specific KoRV subtype contributes to co-infection with chlamydia and associated chlamydial disease progression. One study identified an increase of chlamydial disease in a population of koalas infected with KoRV-B from northern Australia, but total KoRV levels were not associated with chlamydial disease [31]. However, in another study chlamydial disease was found in animals without evidence of KoRV-B infection but testing for other variants was not done [13]. A recent report also did not find an association of KoRV VL with chlamydial disease in a longitudinal study of 16 wild koalas, including in koalas infected with subtypes B, D, and F [15], but the same group reported an association of KoRV-B infection with chlamydial disease in a larger cross-sectional study [14]. Fabijan et al*.* using generic polymerase qPCR reported an association of increasing severity of chlamydial disease with increasing KoRV VLs in koalas from South Australia but not from those in Queensland [32]. They did not report KoRV subtype in this study but in an earlier study they reported no association of KoRV-A infection with chlamydial infection and disease in the same koala population [33]. While it is unclear what role co-infection with chlamydia has on KoRV levels, the qPCR assays we have developed and validated will be useful to further evaluate the role of VLs and any associated immunosuppression in development of chlamydial infection and disease in koalas.

By testing many zoo koalas with known pedigrees, we were able to evaluate potential KoRV transmission routes, which is key for understanding the epidemiology and prevention of these infections. Overall, our results are consistent with the major route of transmission being from dam-to-offspring for KoRV-B, supporting previous findings in a small number of zoo koalas [14, 22]. In our study, we defined joeys as being ≤ one year old compared to Quigley et al*.* who used an age of ≤ 2 years [14], whereas an age cutoff was not provided in the Xu et al*.* report [22]. We did not see a significant difference in transmission rates when we expanded our cutoff to ≤ two years of age. In our study, longitudinal blood specimens were available from five adult, zoo koalas infected with only KoRV-A, but we did not observe new infections in these animals with other KoRV subtypes though their VLs fluctuated over time. In contrast, Quigley et al*.* reported finding four new KoRV-B infections in three adult females and one adult male over a period of almost three years. While they were unable to determine the partner in these wild koalas nor did they report aggressive behavior, the results could suggest sexual transmission since the majority were females, but additional studies are needed to further evaluate KoRV transmission between adults.

Interestingly, in our study we identified eight offspring (7 adults and 1 joey) with discordant KoRV infections from the dam, supporting results from other recent studies [19, 33]. The age at testing for four adults on our study ranged from 25–99 months so they could have acquired their non-A KoRV variants from another koala at the same institution after becoming adults. For example, these koalas were from the Taronga and LA zoos where we found the highest prevalence of KoRV-B and other non-A variants. In addition, the majority were males and could have acquired their infections via male-male aggressive behavior, which is known to increase transmission of other retroviruses like simian immunodeficiency virus (SIV) and simian T-lymphotropic virus (STLV). Quigley et al*.* also reported transmission of KoRV-B between adult koalas but mostly in females more suggestive of transmission during mating [14]. Additional research is required to fully define KoRV transmissibility, including the role of elevated VLs, which will require longitudinal studies.

Vaccinations and antiretroviral drugs have each been proposed as measures to prevent or treat KoRV infection. We show in one leukemic animal with a high viral burden of KoRV-A, -B, and -F that treatment with both raltegravir and tenofovir for 33 days had little effect on reducing VLs and disease outcome. While 33 days is a short period to draw meaningful conclusions on the efficacy of raltegravir and tenofovir in this case, it is noteworthy that both drugs, if active on KoRV, may likely inhibit infection of the exogenously expressed KoRVs because they have been shown to strongly inhibit other gammaretroviruses in vitro [38–40]. It is also unknown how well these antivirals work in vivo, on endogenous KoRV, and if they can reverse cancer. Unlike the related murine leukemia gammaretroviruses, KoRV does not have a cancer inducing oncogene and it is not known if KoRV are integrated in koala genomes near cellular proto-oncogenes to cause insertional mutagenesis [20]. Hence, the mechanism of how KoRV causes cancer is unknown. Interestingly, KoRV-B and KoRV-F have four and five repeats in the U3 region of the long terminal repeat (LTR), respectively, which is involved in gene expression; KoRV-A has only a single copy [7, 22]. In other gammaretroviruses, higher VLs are associated with more copies of these repeats in the U3 region and higher VLs are associated with increased disease and transmissibility of retroviruses [3]. While the extra repeats in the U3 region of KoRV-B may help explain the higher VLs and possible increase in observed neoplasia in these animals, it does not explain the leukemia and lymphoma in the koalas infected with only KoRV-A. Sequence analysis of additional LTRs in KoRV-A animals with and without disease may shed light on this incongruity. Studies of the recently described recombinant KoRVs (recKoRVs) and integration of solo KoRV LTRs near oncogenic transcription sites in increased viral expression and neoplasia in koalas are also needed [20, 21, 41].

Testing and subsequent physical separation of animals infected with KoRV-B from those with only KoRV-A infection or KoRV-negative animals has also been suggested to prevent transmission of these variants and is the current koala management practice in the U.S. However, quarantining non-A KoRV-infected koalas may be impractical and costly, especially for wild populations. Separating koalas infected with specific subtypes could also reduce the koala genetic diversity in zoo-based populations like those in St. Bees Island [37]. A KoRV subtype-generic transmembrane domain of envelope-based vaccine has been developed that induced neutralizing antibodies in vaccinated koalas and reduced plasma VLs by 50–80% in just 12 weeks post vaccination without evidence for vaccine-related pathology [42]. Although the numbers of animals in this study were small and none were infected with KoRV-B to assess effectiveness of the vaccine with this subtype, the results offer a promising prevention and treatment modality if further studies confirm the results.

Our findings also have implications for the biosafety of persons working with koalas. KoRVs are pathogenic and can infect human cell lines in vitro raising questions for potential zoonotic transfer to humans following exposure to zoo or wild koalas. The presence of high levels of the various KoRV-subtypes in multiple tissues, including blood, and the endogenous source of KoRV that cannot be eliminated through breeding, all provide multiple exposure opportunities. While the stability of infectious KoRV in the environment is currently unknown, other gammaretroviruses like feline leukemia virus can survive for at least two days in culture medium but are inactivated after only a few hours if dried [43]. Biosafety practices and procedures for persons handling koalas are important to prevent zoonotic exposures.

There are some limitations of our study. First, our study design was cross-sectional using convenience and archived specimens. Second, we did not test for all known KoRV subtypes or use NGS of PCR products to identify KoRVs. These additional KoRV subtypes were discovered after our study concluded and enough materials and resources were not available to design new assays and re-test koalas for these additional variants. Although NGS is clearly useful for determining the viral composition of a sample, it has not been validated for measuring viral loads and its use with PCR products can lead to overestimation of quasispecies unless corrective measures are taken like the use of unique molecular identifiers [44]. Thirdly, units of pVLs have not been standardized for KoRV quantification making it difficult for interstudy comparisons. We chose to use copies/ug DNA like some groups [18, 30] instead of normalizing by copies of β-actin detected like other groups [8, 13, 16, 33], since β-actin can be present in many copies/genome and exists as many pseudogenes unlike single copy genes per haploid genome like the RNase P protein subunit p30 (RPP30) typically used for pVLs in HTLV infection [45]. The complete koala genome was not available at the time of our study for identifying primers for pVL normalization with single copy genes. Fourthly, as with other molecular epidemiologic studies using PCR, our results could include detection of defective genomes and may not reflect infectivity of the detected variants [7, 16, 20, 46]. Finally, as koalas are infected with multiple subtypes, determination of the contributions of a single or multiple KoRV variants to neoplasia or other diseases is complex and requires research beyond the scope of our study, including animal model studies with infectious molecular clones.

## Conclusions

Our results show a significant association of non-A KoRV infection and increased plasma VLs with leukemia and other cancers in infected koalas. We also document carcinoma in koalas infected with only KoRV-A. Although we confirm dam-to-offspring transmission of these non-A KoRV variants, we also show other routes are possible. Our validated qPCR assays will be useful to further characterize KoRV epidemiology and its zoonotic potential.

## Supplementary information

**Additional file 1:** Additional table and figure.

## Data Availability

The datasets used and analyzed during our study are available from the corresponding author on reasonable request.

## Abbreviations

KoRV: Koala retrovirus
VL: Viral load
pVL: Proviral load
qPCR: Quantitative PCR
PCR: Polymerase chain reaction
HIV: Human immunodeficiency virus
GaLV: Gibbon ape leukemia virus
HTLV-1: Human T-lymphotropic virus type 1
THTR1: Thiamine transport protein 1
Env: Envelope protein
LA: Los Angeles
IACUC: Institutional Animal Care and Use Committee
PBMCs: Peripheral blood mononuclear cells
gDNA: Genomic DNA
Cq: Quantification
R^2^: Correlation coefficient
BLD: Below the assay detection limit
pt: Post treatment
RecKoRV1: Recombinant KoRVs
LTR: Long terminal repeat
NGS: Next generation sequencing
RPP30: RNase P protein subunit p30
SIV: Simian immunodeficiency virus
STLV: Simian T-lymphotropic virus
