# Supplementary material for: Koala retrovirus diversity, transmissibility, and disease associations
Source: Retrovirology. 2020 Oct 2;17:34. doi: 10.1186/s12977-020-00541-1 (PMC7530975; doi:10.1186/s12977-020-00541-1)

**Additional Material**

**Table S1. Koala retrovirus (KoRV) envelope (env) generic and type-specific and koala ß-actin quantitative PCR (qPCR) primers and probes**

**__________________________________________________________________________________________________________________________________**

**Assay Forward primers (5’ to 3’) TaqMan probes (5’ to 3’) Reverse primers (5’ to 3’) __________________________________________________________________________________________________________________________________**

**KoRV generic^1^** CACCCAGGYGTGCAGTTG ATCGGGGCCCASTAGTACCGGTGGGGGRCTTGTGA TGGGAGGTCCTTGTYCTGCGAGGA CAGGGAGACCTTGTACTACAAGGAC

**KoRV-A** GCCAGGCCCCCTGATTCAA AGCTACATCCCAGGGTTCCCCAAGTGATCTGATTATAAGCATG GCACACGTAGAACTGGGACC

**KoRV-B** GCCAGAATCTCAACAGTCTGC AGGTTCCATAGCTCGATTGCCTGACCAACCCTCTGCCGACCT GGGACACACATAGAACTGAGATTG

**KoRV-E** GGCCAGGCCACCTCTGTTT TAGGGTTCCCCCCCATCCGGAGGCTGCAAAGCCAG CTCCGGGCCTCGGACAA

**KoRV-F** GGCCAGGCCGTCTTTGCA TCCTCCTCCCCACCCAGTGGCTGTAAAGCCAGGCC GGGACATAGCAATTCTGGTTCGA

**KoRV-J** GCCGAAATGAACCCTCACCG TCCCAAACGACACTTCCCGTCTGGGACAGT CAGGGTGGCTCGACTGCTTC

**koala β-actin** GCAAAGCTGGCTTTGCAGGA ACCATCACACCCTGATGTCTGGGGCGCCCCACA CATCCCCCACGTAGCTGTCT

**__________________________________________________________________________________________________________________________________**

1. The generic KoRV qPCR assay utilizes two reverse primers for optimal sensitivity.

**Additional Figure legend**

**S1. Longitudinal koala retrovirus (KoRV) subtype levels in a koala with leukemia and treated with antiretroviral therapy.** Log10 KoRV levels in genomic DNA (copies/ug) from peripheral blood mononuclear cells (upper panel) and plasma RNA (copies/mL; lower panel). Koala was given integrase (raltegravir) and nucleotide reverse transcriptase inhibitors (tenofovir) at day zero and whole blood samples were collected every 3 – 4 days. KoRV levels below the level of detection or the estimated assay cut-offs were set to zero for the analyses. Samples with test results below the limit of detection were not included in the plots.


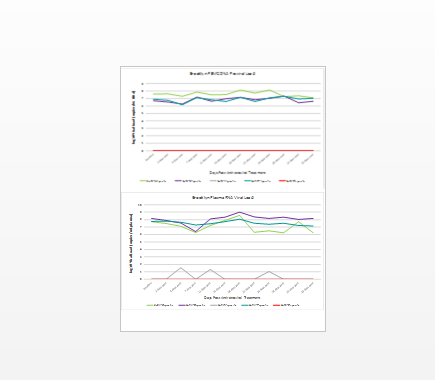

Supplement: Supplementary file 1 — Additional file 1: Additional table and figure. [file 12977_2020_541_MOESM1_ESM.docx]
